# Communication channels for receiving air quality alerts among adults in the United States

**DOI:** 10.1016/j.pmedr.2021.101677

**Published:** 2021-12-23

**Authors:** Lindsay K. Tompkins, Audrey F. Pennington, Kanta D. Sircar, Maria C. Mirabelli

**Affiliations:** a Epidemic Intelligence Service, Center for Surveillance, Epidemiology and Laboratory Services, Centers for Disease Control and Prevention, Atlanta, GA, United Sates; b Asthma and Community Health Branch, Division of Environmental Health Science and Practice, National Center for Environmental Health, Centers for Disease Control and Prevention, Atlanta, GA, United Sates

**Keywords:** Air pollution, Air quality awareness, Respiratory disease, Heart disease, Health communication, Environmental health, Environmental exposure

## Abstract

Exposure to air pollution is associated with respiratory and cardiovascular effects, particularly among people with underlying respiratory and heart disease. It is therefore important for individuals with respiratory and heart disease to be aware of air quality. However, information about the most effective communication channels for disseminating air quality alerts is limited. We assessed communication channels used for receiving air quality alerts among U.S. adults using data from the summer 2020 wave of *ConsumerStyles*, a nationally representative survey of U.S. adults (n = 4053). We calculated weighted percentages of respondents who received air quality alerts from six communication channels and stratified by demographic and health characteristics. We calculated weighted prevalence ratios (PRs) adjusted for sex, age, race/ethnicity, and education to assess if communication channel use varied by presence of respiratory or heart disease. Sixty-four percent of U.S. adults had heard or read about air quality alerts. Television was the most commonly reported communication channel for receiving alerts (57.5%), followed by app on mobile phone or device (30.2%) and internet or social media (26.4%). Communication channels differed most prominently by age. The proportion of adults receiving alerts from specific communication channels did not notably vary by presence of heart disease. Adults with respiratory disease more often reported receiving alerts from their doctor’s office than adults without respiratory disease (PR: 3.10, 95% confidence interval: 1.49, 6.45). These findings can be used by public health officials to increase awareness of poor air quality days and improve the reach of alerts to target populations.

## 1 Introduction

Exposure to air pollution is associated with respiratory and cardiovascular effects, including increases in emergency department visits, hospital admissions, and mortality, particularly among individuals with underlying respiratory and heart disease (Brook et al., 2004, Brook et al., 2010, Tiotiu et al., 2020, U.S. Environmental Protection Agency, 2019). In addition, air pollution was the fourth leading risk factor for death globally and contributed to more than 6.6 million deaths worldwide in 2019 (Health Effects Institute, 2020). It is therefore important for individuals with respiratory and heart disease to be aware of air quality, understand the risks associated with exposure to air pollution, and know how to reduce exposure to air pollution. This awareness is particularly important during extreme air pollution events such as wildfires, which are increasing in frequency and impacting larger geographic areas as the climate warms (U.S. Global Change Research Program, 2018). Timely communication of air quality alerts coupled with exposure-reducing strategies may lessen the substantial health and economic burdens related to exposure to air pollution (Rappold et al., 2014).

Recent information about which communication channels are most effective for disseminating air quality alerts is limited. We aimed to build on a previous analysis of 2014 population-based *ConsumerStyles* survey data that explored how U.S. adults receive air quality alerts (Pennington et al., 2019). In this analysis by Pennington et al., television was the most common communication channel for receiving these alerts, while alerts via digital media channels such as the internet and apps on a mobile phone or device reached fewer than one-fifth of adults who had received air quality alerts. The summer wave of the 2020 *ConsumerStyles* survey repeated the question on communication channels used to receive air quality alerts to provide updated information on how U.S. adults receive this information. We aimed to reassess the communication channels that U.S. adults use to receive air quality alerts and to explore differences in communication channels used by demographic and health characteristics.

## 2 Methods

### 2.1 Survey methods

We analyzed data from the summer wave of the 2020 ConsumerStyles survey. ConsumerStyles is a cross-sectional survey conducted by Porter Novelli Public Services (Washington, DC). Respondents to the spring wave of the 2020 survey (SpringStyles) were invited to participate in the summer wave (SummerStyles). SpringStyles was conducted using Ipsos’ KnowledgePanel®, an online panel that is representative of the non-institutionalized U.S. population. The panel maintains approximately 60,000 panelists who were randomly recruited by mail using probability-based sampling.

The 2020 SpringStyles survey was sent to 11,097 panelists and completed by 6,463 (58.2%). The 2020 SummerStyles was completed by 4,053 (62.7%) SpringStyles respondents in June 2020.

Data were weighted using sex, age, household income, race/ethnicity, household size, education, census region, metro status, and parental status of children 12–17 years old to match U.S. Current Population Survey proportions.

This activity was reviewed and determined to be exempt from full institutional review board review at the Centers for Disease Control and Prevention because of its use of de-identified secondary data.

### 2.2 Measures

Respondents were asked “Have you ever heard or read about the Air Quality Index or air quality alerts where you live?” If respondents answered yes, they were then asked, “Where did you hear or read about air quality alerts?” Response options were radio, television, newspaper, internet or social media, app on mobile phone or device, doctor’s office, and don’t know. Respondents could select multiple options. Respondents’ demographic (individual- and household-level) and health (respiratory and heart disease during the past year) characteristics were available.

### 2.3 Statistical analysis

We calculated weighted percentages and 95% confidence intervals (CIs) for each communication channel used to receive air quality alerts, overall and by demographic and health characteristics. We also calculated prevalence ratios (PRs) using predicted marginal probabilities from logistic regression models (Bieler et al., 2010) to assess whether communication channels used for receiving air quality alerts varied by presence of respiratory or heart disease after adjusting for age, sex, race/ethnicity, and education. We used survey weights provided with the SummerStyles data to generate results that are representative of the U.S. adult population. Analyses were conducted in SAS 9.4 (SAS Institute, Inc., Cary, North Carolina) and SAS-callable SUDAAN (RTI International, Research Triangle Park, North Carolina).

## 3 Results

An estimated 63.5% (95% CI: 61.6, 65.3) of U.S. adults reported hearing or reading about the Air Quality Index or air quality alerts. Responses regarding hearing or reading about alerts did not differ by respondents’ health status (heard or read about alerts: respiratory disease 66.4% [95% CI: 60.6, 72.1], no respiratory disease 63.2% [95% CI: 61.2, 65.2]; heart disease 69.4% [95% CI: 60.5, 78.3], no heart disease 63.3% [95% CI: 61.4, 65.2]). Characteristics of the 2,782 respondents who heard or read about alerts are shown in Table 1. Age ranged from 18 to 94 years. Nearly 10% reported having a respiratory disease (i.e., asthma, emphysema/chronic obstructive pulmonary disease [COPD]) and 2.9% reported having heart disease (i.e., atrial fibrillation, congestive heart failure, angina, heart attack) during the past year.

**Table 1:** Demographic and health characteristics of U.S. adults who heard or read about the Air Quality Index or air quality alerts — *ConsumerStyles*, 2020.

**Characteristic**	**Number of Respondents**^a^	**Weighted % (95% CI)**
**Individual characteristics**		
Age, years		
18–29	210	17.0 (14.7, 19.3)
30–44	609	23.5 (21.6, 25.4)
45–59	862	26.6 (24.7, 28.4)
60–74	859	25.7 (24.0, 27.4)
≥75	242	7.2 (6.3, 8.2)
Sex		
Male	1468	50.3 (48.1, 52.5)
Female	1314	49.7 (47.5, 51.9)
Race/Ethnicity		
White, non-Hispanic	2124	66.9 (64.6, 69.2)
Black, non-Hispanic	171	9.1 (7.6, 10.5)
Other, non-Hispanic	232	9.2 (7.7, 10.6)
Hispanic	255	14.9 (13.0, 16.8)
Education		
High school or less	709	30.9 (28.7, 33.1)
Some college	785	28.7 (26.6, 30.7)
Bachelor’s degree or higher	1288	40.5 (38.3, 42.6)
Smoking status		
Current smoker	222	8.6 (7.3, 9.8)
Former smoker	835	26.5 (24.6, 28.3)
Lifetime non-smoker^b^	1725	65.0 (62.9, 67.0)

**Health characteristics**		
Respiratory disease^c^		
No	2519	90.1 (88.7, 91.5)
Yes	263	9.9 (8.5, 11.3)
Heart disease^d^		
No	2682	97.1 (96.4, 97.7)
Yes	100	2.9 (2.3, 3.6)

**Household characteristics**		
Household income		
< $25,000	197	9.8 (8.2, 11.4)
$25,000 to < $50,000	386	14.8 (13.1, 16.4)
$50,000 to < $75,000	465	16.8 (15.1, 18.5)
≥ $75,000	1734	58.6 (56.3, 60.9)
U.S. census region		
Northeast	506	18.6 (16.8, 20.4)
Midwest	598	20.2 (18.4, 21.9)
South	924	34.4 (32.2, 36.5)
West	754	26.9 (24.9, 28.9)

Percentages are weighted to the U.S. Current Population Survey.

a Unweighted sample size.

b Includes individuals with unknown smoking status.

c Includes asthma and emphysema/chronic obstructive pulmonary disease (COPD) during the past year.

d Includes atrial fibrillation, congestive heart failure, or other heart condition (angina or heart attack) during the past year.

Television was the most commonly reported communication channel used for receiving air quality alerts (57.5%) among adults who had received alerts (Table 2). App on mobile phone or device (30.2%) and internet or social media (26.4%) were reported by more than one-quarter of respondents. Few respondents reported hearing or reading about air quality alerts at their doctor’s office (2.3%). Only 4.1% of respondents did not know where they heard or read about air quality alerts. Over one-third of respondents reported receiving alerts from more than one channel (38.8%, 95% CI: 36.7, 41.0).

**Table 2:** Weighted percentages of U.S. adults who reported communication channels for receiving air quality alerts, stratified by demographic and health characteristics — *ConsumerStyles*, 2020.

	**Communication Channel**					
	**Television**	**App on mobile phone or device**	**Internet or social media**	**Radio**	**Newspaper**	**Doctor’s office**
	**Weighted % (95% CI)**	**Weighted % (95% CI)**	**Weighted % (95% CI)**	**Weighted % (95% CI)**	**Weighted % (95% CI)**	**Weighted %(95% CI)**
**Total**	57.5 (55.2, 59.7)	30.2 (28.0, 32.3)	26.4 (24.4, 28.5)	19.4 (17.7, 21.0)	16.0 (14.4, 17.5)	2.3 (1.6, 3.0)
**Individual characteristics**						
Age, years						
18–29	29.5 (22.2, 36.8)	44.8 (37.0, 52.6)	34.0 (26.8, 41.3)	12.3 (7.1, 17.6)	11.5 (6.4, 16.6)	1.6 (0.0, 3.5)
30–44	45.7 (41.2, 50.2)	40.9 (36.5, 45.3)	34.6 (30.3, 39.0)	13.7 (10.7, 16.7)	10.4 (7.6, 13.1)	2.6 (1.0, 4.2)
45–59	60.3 (56.5, 64.0)	25.7 (22.4, 29.0)	24.4 (21.0, 27.7)	24.7 (21.5, 27.9)	13.3 (10.8, 15.8)	2.2 (1.0, 3.3)
60–74	77.8 (74.7, 80.8)	19.1 (16.3, 22.0)	18.8 (15.8, 21.8)	23.6 (20.7, 26.6)	21.8 (18.8, 24.7)	2.9 (1.5, 4.2)
≥75	78.9 (73.3, 84.6)	16.5 (11.8, 21.3)	16.8 (11.7, 22.0)	19.3 (14.2, 24.4)	33.6 (27.3, 40.0)	1.5 (0.0, 3.0)
Sex						
Male	59.6 (56.5, 62.8)	28.0 (25.0, 31.0)	27.9 (25.0, 30.8)	21.7 (19.3, 24.1)	16.4 (14.3, 18.5)	1.6 (0.9, 2.2)
Female	55.3 (52.0, 58.5)	32.3 (29.3, 35.4)	25.0 (22.1, 27.8)	17.0 (14.7, 19.3)	15.5 (13.2, 17.8)	3.1 (1.9, 4.3)
Race/Ethnicity						
White, non-Hispanic	57.8 (55.3, 60.3)	30.5 (28.1, 32.9)	25.0 (22.8, 27.2)	21.1 (19.1, 23.1)	15.9 (14.2, 17.7)	2.1 (1.4, 2.9)
Black, non-Hispanic	32.6 (24.1, 41.1)	27.4 (19.6, 35.2)	19.6 (13.3, 25.9)	20.1 (13.5, 26.6)	19.4 (12.9, 25.8)	3.8 (0.4, 7.2)
Other, non-Hispanic	43.4 (35.3, 51.5)	31.3 (23.6, 39.0)	40.2 (31.9, 48.5)	15.3 (10.2, 20.4)	15.1 (9.6, 20.5)	2.3 (0.6, 4.1)
Hispanic	58.5 (51.5, 65.6)	29.6 (23.1, 36.1)	28.8 (22.4, 35.3)	13.4 (9.3, 17.6)	14.6 (9.7, 19.5)	2.2 (0.1, 4.2)
Education						
High school or less	67.0 (62.6, 71.4)	22.7 (18.6, 26.8)	19.6 (16.0, 23.3)	14.5 (11.5, 17.6)	11.0 (8.2, 13.8)	3.0 (1.5, 4.6)
Some college	57.6 (53.1, 62.1)	31.9 (27.7, 36.1)	23.7 (19.8, 27.7)	18.0 (14.9, 21.1)	16.1 (13.0, 19.3)	2.4 (1.2, 3.6)
Bachelor’s degree or higher	50.1 (47.0, 53.2)	34.6 (31.6, 37.6)	33.6 (30.5, 36.6)	24.0 (21.4, 26.6)	19.6 (17.2, 22.0)	1.7 (0.8, 2.6)
Smoking status						
Current smoker	60.3 (52.7, 68.0)	27.4 (20.7, 34.1)	24.9 (18.2, 31.6)	15.8 (10.7, 21.0)	11.9 (7.0, 16.9)	3.4 (0.6, 6.3)
Former smoker	67.5 (63.8, 71.1)	24.9 (21.7, 28.1)	21.3 (18.2, 24.5)	19.6 (16.7, 22.5)	16.5 (13.6, 19.5)	2.2 (1.1, 3.4)
Lifetime non-smoker^a^	53.0 (50.1, 56.0)	32.7 (29.8, 35.5)	28.7 (26.0, 31.4)	19.7 (17.5, 21.9)	16.3 (14.3, 18.3)	2.2 (1.3, 3.1)

**Health characteristics**						
Respiratory disease^b^						
No	57.5 (55.2, 59.9)	29.9 (27.7, 32.1)	26.0 (23.9, 28.1)	19.7 (17.9, 21.4)	15.9 (14.3, 17.6)	1.9 (1.3, 2.5)
Yes	56.9 (49.3, 64.5)	32.8 (25.6, 40.0)	30.4 (23.2, 37.5)	16.5 (11.7, 21.2)	16.1 (10.5, 21.7)	6.4 (2.3, 10.5)
Heart disease^c^						
No	57.0 (54.7, 59.3)	30.4 (28.2, 32.5)	26.8 (24.7, 28.9)	19.2 (17.5, 20.9)	15.7 (14.1, 17.3)	2.3 (1.6, 3.0)
Yes	72.3 (62.4, 82.2)	23.3 (13.9, 32.7)	14.7 (7.9, 21.4)	23.2 (13.9, 32.4)	25.3 (16.0, 34.6)	3.4 (0.0, 7.5)

**Household characteristics**						
Household income						
< $25,000	57.2 (48.4, 66.0)	30.6 (22.4, 38.9)	30.6 (22.5, 38.8)	13.0 (7.9, 18.2)	13.8 (8.1, 19.6)	4.0 (0.2, 7.7)
$25,000 to < $50,000	59.3 (53.1, 65.4)	28.5 (22.8, 34.2)	21.2 (16.3, 26.0)	12.1 (8.6, 15.7)	12.2 (8.4, 16.0)	4.1 (1.7, 6.4)
$50,000 to < $75,000	57.7 (52.2, 63.3)	31.4 (26.0, 36.7)	25.0 (20.2, 29.7)	15.2 (11.8, 18.5)	13.7 (10.3, 17.2)	2.7 (1.0, 4.3)
≥ $75,000	57.0 (54.2, 59.8)	30.2 (27.6, 32.8)	27.5 (24.9, 30.1)	23.4 (21.1, 25.8)	17.9 (15.8, 20.0)	1.5 (0.9, 2.1)
U.S. census region						
Northeast	53.9 (48.5, 59.3)	32.5 (27.2, 37.8)	24.5 (19.7, 29.2)	16.4 (12.6, 20.2)	18.0 (14.0, 22.0)	2.2 (0.7, 3.8)
Midwest	59.0 (54.2, 63.8)	34.3 (29.8, 38.9)	22.1 (18.0, 26.2)	20.3 (16.6, 24.0)	12.8 (9.8, 15.9)	1.0 (0.0, 2.0)
South	61.5 (57.7, 65.3)	28.2 (24.7, 31.8)	26.6 (23.1, 30.1)	19.5 (16.6, 22.4)	13.8 (11.2, 16.4)	1.4 (0.6, 2.3)
West	53.7 (49.3, 58.0)	27.9 (23.9, 31.9)	30.9 (26.8, 34.9)	20.5 (17.3, 23.7)	19.7 (16.5, 22.9)	4.5 (2.6, 6.3)

Percentages are weighted to the U.S. Current Population Survey.

a Includes individuals with unknown smoking status.

b Includes asthma and emphysema/chronic obstructive pulmonary disease (COPD) during the past year.

c Includes atrial fibrillation, congestive heart failure, or other heart condition (angina or heart attack) during the past year.

When examining differences by demographic characteristics, television was the most widely reported communication channel in nearly all groups (Table 2). Reports of receiving air quality alerts via television increased by age group and decreased by education level. The one group that did not most commonly report receiving alerts via television was the 18–29-year-olds, who more often reported using an app on their mobile phone or device. Among non-Hispanic Black adults and 30–44-year-olds, the proportion receiving alerts via an app on a mobile phone or device was similar to the proportion receiving alerts via television (Black, non-Hispanic: 32.6% [95% CI: 24.1, 41.1] [television], 27.4% [95% CI: 19.6, 35.2] [app]; 30–44-year-olds: television 45.7% [95% CI: 41.2, 50.2] [television], 40.9% [95% CI: 36.5, 45.3] [app]).

Communication channels differed most prominently by age group. For example, receiving alerts via app on mobile phone or device was most common among those under the age of 45 years (reported by more than 40%), whereas fewer than 20% of adults 60 years of age and older received alerts through this channel.

When examining differences in communication channel use by health characteristics, the proportion of adults receiving alerts from television, app on mobile phone or device, internet or social media, radio, and newspaper did not notably vary by presence of respiratory or heart disease (Table 2). Adults with respiratory disease more often reported receiving air quality alerts at their doctor’s office compared with adults without respiratory disease (6.4% vs. 1.9%, respectively). This difference remained after adjusting for age, sex, race/ethnicity, and education (PR: 3.10 [95% CI: 1.49, 6.45]). When adjusting for covariates, there was no association between presence of respiratory or heart disease and the other communication channels. Associations between respiratory disease and other communication channels for receiving alerts were: television (PR: 0.97 [95% CI: 0.85, 1.10]), app on mobile phone or device (PR: 1.13 [95% CI: 0.90, 1.41]), internet or social media (PR: 1.26 [95% CI: 1.00, 1.59]), radio (PR: 0.92 [95% CI: 0.69, 1.24]), newspaper (PR: 1.04 [95% CI: 0.73, 1.50]). Associations between heart disease and communication channels for receiving alerts were: television (PR: 1.02 [95% CI: 0.84, 1.23]), app on mobile phone or device (PR: 1.06 [95% CI: 0.75, 1.50]), internet or social media (PR: 0.71 [95% CI: 0.45, 1.12]), radio (PR: 1.02 [95% CI: 0.66, 1.59]), newspaper (PR: 1.19 [95% CI: 0.77, 1.83]), doctor’s office (PR: 1.76 [95% CI: 0.50, 6.27]).

## 4 Discussion

Air quality alerts on television reached the largest percentage of U.S. adults, reaching more than half of U.S. adults aware of air quality alerts. This is consistent with findings from 2014 *ConsumerStyles* data, although the proportion reporting this channel decreased from 76% to 58% (Pennington et al., 2019). Declines in proportions receiving air quality alerts via television were especially pronounced for certain demographic groups, including non-Hispanic Black adults; among this group, the proportion receiving alerts via television decreased from 91% in 2014 (Pennington et al., 2019) to 33% in 2020. In contrast, we found that air quality alerts via an app on a mobile phone or device reached the second largest percentage of U.S. adults at 30% in 2020 compared with less than 6% in 2014 (Pennington et al., 2019). In 2020, the proportion of 18- to 29-year-olds using an app on their mobile phone or device to receive alerts surpassed the proportion using television, and proportions of non-Hispanic Black adults and 30- to 44-year-olds using an app to receive alerts were similar to proportions using television. These findings follow national trends in television use and smartphone ownership among adults, with adults who receive television via cable or satellite decreasing from 76% in 2015 to 56% in 2021 (Pew Research Center, 2021a, Pew Research Center, 2021) and smartphone ownership among adults increasing from 55% in 2014 to 85% in 2021 (Pew Research Center, 2021a). Smartphone ownership is particularly high among adults less than 50 years old, with over 95% owning a device, compared to 61% of those 65 years and older (Pew Research Center, 2021b), which also corresponds with our finding that the proportion of adults receiving air quality alerts via an app on a mobile phone or device decreases as age increases. The growth in smartphone ownership, and specifically the increased access to smartphone applications that communicate air quality information, may provide opportunities for more frequent communication of real-time air quality alerts that are specific to the user’s geographic location and strategies for reducing exposure (Rappold et al., 2019).

Similar to the findings of the analysis using 2014 *ConsumerStyles* data (Pennington et al., 2019), we observed more variation in communication channels used to receive air quality alerts by demographic characteristics than by health characteristics. Compared with adults without respiratory disease, adults with respiratory disease more often reported receiving air quality alerts from their doctor's office. With only 2.3% of adults aware of air quality alerts reporting this channel, doctor's office was not a commonly reported communication channel for receiving air quality alerts. However, this was the only communication channel that differed by health characteristic. Therefore, our findings continue to support that using demographic characteristics, such as age and race/ethnicity, may be a more effective strategy for optimizing reach of air quality alerts to specific groups than using health characteristics. For instance, results of communication channels used to receive alerts by racial and ethnic status can be used to reach non-Hispanic Black adults, a group with a higher prevalence of asthma (U.S. Centers for Disease Control and Prevention, 2021) and therefore at greater risk for health effects related to exposure to poor air quality (Tiotiu et al., 2020, U.S. Environmental Protection Agency, 2019); our results suggest that air quality alerts via television or mobile apps may best reach this demographic group. Our findings also highlight rarely used communication channels; increasing use of these channels may help alerts reach new populations. For example, opportunities exist for expanding the reach of air quality health risk communication to patients with respiratory and heart disease through their healthcare providers; considerations for provider dissemination of information on air quality and associated health risks in the cardiac rehabilitation setting have been described by Hano et al. (2019).

A strength of this analysis is that we used data representative of the U.S. adult population; proportions of adults with past-year histories of respiratory and heart disease were similar to other representative surveys (U.S. Centers for Disease Control and Prevention, 2021, Mirabelli et al., 2018). Limitations include the use of self-reported data and the lack of information on specific communication platforms used to receive alerts, which communication channels respondents preferred to use to receive alerts, and how frequently respondents accessed each communication channel. Additional information on these characteristics of communication channel use could help inform communication strategies and focus dissemination efforts to improve reach of air quality alerts.

Our findings provide updated information on how U.S. adults learn about air quality alerts. These results can be used by public health officials interested in increasing awareness of poor air quality days to improve the reach of alerts to target populations, thereby reducing exposure to air pollution and attenuating associated adverse health effects.

## Funding

This research did not receive any specific grant from funding agencies in the public, commercial, or not-for-profit sectors.

Financial Disclores

The authors have no financial relationships relevant to this article to disclose.

## Declaration of Competing Interest

The authors declare that they have no known competing financial interests or personal relationships that could have appeared to influence the work reported in this paper.
